# Enhancing quality of life in head and neck cancer patients: a comparative analysis of 3D exoscope-assisted surgery vs. traditional approaches

**DOI:** 10.3389/fsurg.2024.1358500

**Published:** 2024-04-16

**Authors:** Filippo Marchi, Elisa Bellini, Alessandro Ioppi, Federica Simoni, Andrea Iandelli, Marta Filauro, Francesco Mora, Claudio Sampieri, Giorgio Peretti

**Affiliations:** ^1^ Unit of Otorhinolaryngology-Head and Neck Surgery, IRCCS Ospedale Policlinico San Martino, Genoa, Italy; ^2^ Department of Surgical Sciences and Integrated Diagnostics (DISC), University of Genova, Genoa, Italy; ^3^ Department of Otorhinolaryngology, Head and Neck Surgery, IRCCS Ospedale Policlinico San Martino, Genoa, Italy; ^4^ Department of Otorhinolaryngology, Head and Neck Surgery, “S. Chiara” Hospital, Azienda Provinciale Per I Servizi Sanitari (APSS), Trento, Italy; ^5^ Department of Experimental Medicine (DIMES), University of Genoa, Genoa, Italy; ^6^ Department of Otolaryngology, Hospital Cliníc, Barcelona, Spain; ^7^ Functional Unit of Head and Neck Tumors, Hospital Cliníc, Barcelona, Spain

**Keywords:** quality of life, oropharyngeal cancer, laryngeal cancer, dysphagia, dysphonia, exoscopic surgery, head and neck cancer, transoral surgery

## Abstract

**Introduction:**

The increasing population of survivors of head and neck carcinomas is becoming more conspicuous. Consequently, the pivotal role of quality of life, particularly elucidated through the assessment of dysphagia and dysphonia, is progressively influencing the decision-making process. The current study aims to assess whether VITOM 3D could offer a comparable post-treatment quality of life to traditional approaches for patients with laryngeal cancer and oro-hypopharyngeal cancer.

**Methods:**

A case series of laryngeal cancer and oro-hypopharyngeal cancer patients treated either with an exoscopic-assisted surgical setup and with conventional treatments (transoral microsurgery and radio-chemotherapy) at the Otolaryngology Unit of IRCCS San Martino Hospital, Genoa, is presented. The post-treatment quality of life of the two cohorts were compared through the administration of the University of Washington Quality of Life Questionnaire, Voiceik Handicap Index-10, M.D. Anderson Dysphagia Inventory were administrated to both cohorts of patients.

**Results:**

In the laryngeal cancer group, a total of 79 patients were included. Of these, 50.1% underwent transoral exoscope-assisted surgery, while 49.9% underwent primary transoral microscopic-assisted surgical approach. No significant differences were observed in terms of the University of Washington Quality of Life Questionnaire and Voice Handicap Index-10 between the two subgroups. Conversely, in the oro-hypopharyngeal cancer group, 43 patients were included. Of these, 37.2% underwent primary transoral exoscope-assisted surgery, while 62.8% received (chemo)radiotherapy. No notable differences were reported in terms of the University of Washington Quality of Life Questionnaire and M.D. Anderson Dysphagia Inventory between the transoral exoscope-assisted surgery and (chemo)radiotherapy subgroups.

**Conclusions:**

Assessments of quality of life, conducted through the University of Washington Quality of Life Questionnaire questionnaire, dysphonia evaluations using the Voice Handicap Index-10, and dysphagia assessments employing the M.D. Anderson Dysphagia Inventory questionnaire, demonstrate analogous outcomes between conventional treatment modalities and transoral interventions utilizing the 3D exoscope.

## Introduction

The increasing population of head and neck cancer (HNC) survivors is partly due to improved treatment and changing epidemiology, with Human Papillomavirus (HPV)-associated cases having a more favorable prognosis than non-HPV related ones (1). This leads to long-lasting cancer-related effects that extend beyond the primary treatment phase (2–5), ultimately diminishing the health-related quality of life (QOL) compared to other types of cancer occurring in different anatomical sites. In addition to general complaints such as pain and fatigue, HNC patients often grapple with issues like oral dysfunction, voice, speech and swallowing problems with related social withdrawal and emotional distress (1, 6–8).

While the treatment of any malignancy traditionally prioritizes disease control, particularly local or regional control, with an emphasis on extended survival as the ultimate goal, more recent times have seen quality of life recognized as a critical factor in the decision making process (9, 10).

In oropharyngeal cancer (OPC), dysphagia has been strongly linked to a lower QOL and can be assessed using various questionnaires, such as the M.D. Anderson Dysphagia Inventory (MDADI) (5, 11–13).

On the other hand, the Voice Handicap Index-10 (VHI-10) (14) is a widely used patient perceptual questionnaire that evaluates the impact of voice problem on an individual's life in patient affected by laryngeal cancer (LC).

Informed decisions about future treatment options require knowledge of how contemporary therapies affect life of HNC patients (15).

In the recent times, there has been a surge in technological innovations aimed at improving patients' QOL (16).

Lately, various devices, including exoscopes like VITOM 3D exoscope (Karl Storz—Tuttlingen, Germany), which are high-definition cameras equipped with optical and digital zoom capabilities and paired with a light source capable of providing 3D vision, have been developed (17–20). This technology was recently introduced in the field of otorhinolaryngology (21–24).

The exploration of emerging technologies should prioritize “patients” outcomes’ considerations instead of embracing technology merely for the sake of it. Within the realm of technological advancements, certain decisions are straightforward and align with natural evolution, whereas others necessitate a thorough analysis of tangible benefits.

The aim of the current study is to assess whether exoscopes could represent a valuable option in terms of improving the quality of life for LC and OPC or hypopharyngeal (HPC) patients when compared to well-established treatments.

## Materials and methods

We retrospectively analyzed two groups of patients who were referred to the Otorhinolaryngology unit of S. Martino Hospital in Genoa from February 2019 to January 2023, affected by LC or OPC/HPC. The study was conducted in accordance with the Declaration of Helsinki and was approved by the local Ethics Committee (CER Liguria: 230/2019). All the cases in the study underwent multidisciplinary discussion and were directed towards first-line treatments, which could be either surgical or non-surgical. Surgical options included Transoral exoscopic laser surgery (TOELS) for OPC, and (Transoral laser microsurgery) TOLMS or TOELS for LC, while non-surgical approaches involved (chemio)radiotherapy [(CT)RT] for OPC. In the TOELS group, the exoscope was applied, equipped with a 3D-HD camera and ARTipTM robotic cruise system, allowing manual control via a joystick or robotic arm. It offers features like zoom, focus, illumination, and horizontal alignment, producing either 4K or 3D images displayed on a 3D-HD screen. Stereoscopic image processing is facilitated by polarized glasses worn by operators and operating room staff.

Subsequently, we administered various types of questionnaires to the patients to assess their quality of life, voice, and dysphagia.

### Laryngeal cancer group (LC)

Patients affected by LC and treated either using a transoral exoscope-assisted surgical approach (TOELS) or a microscopic-assisted surgical approach (TOLMS) were included. Inclusion criteria were: (1) early-intermediate suspicious or malignant glottic lesions classified as cT1- cT2 N0 with the 8th version of the TNM classification system (25); (2) good predicted laryngeal exposure according to the “Laryngoscore” exposure score (26) and (3) patients who have not undergone prior surgical or radiation treatments. Patients' demographics, tumor features, duration of surgical procedure and surgical outcomes (hospital stay and margins status) were collected. We have decided to explore vocal outcomes as a means of assessing the quality of life in patients. All patients underwent the VHI-10 to examine vocal quality within 2 weeks before and, at least, 3 months after treatment. The VHI-10 represents a streamlined yet equally comprehensive alternative to the VHI-30, with a specific focus on assessing the impact of treatment on voice and, by extension, the associated quality of life. Given that alterations in voice are a primary concern in early-stage laryngeal tumors, we have selected a questionnaire tailored to evaluate this particular domain. VHI-10 questionnaire consists of a 10-item scale, each item being scored between 0 (never) and 4 (always). A score smaller or equal to 10 represents little or no handicap caused by dysphonia, a 10 to 20 score characterizes a medium handicap and a score equal or greater than 20 describes a severe QOL impact in terms of dysphonia.

### Oropharyngeal cancer group (OPC/HPC)

This group of patients consists of subjects affected by an OPC/HPC, classified as cT1-3 N0-2 with the 8th version of the TNM classification system (25). Patients were treated by means of TOELS and (CT)RT. In patients undergoing TOELS, a good exposure of the oropharyngeal corridor, with a surgical light greater than or equal to 80% was predicted using the pharyngoscore (27). Patients' demographics and tumor features were collected. We have decided to explore dysphagia outcomes as a means of assessing the quality of life in patients. All patients were investigated through the MDADI that is a self-administered questionnaire for evaluating the impact of dysphagia within 1 months before and 3 months after treatment(s).

In details, The MDADI stands out as one of the most frequently utilized questionnaires for assessing the quality of life in patients affected with oropharyngeal tumors. Primarily designed to delve into the quality of life associated with swallowing function— a primary function often impacted by oropharyngeal tumors—it serves as a valuable tool in evaluating patient well-being.

The questionnaire has four subdomains: global, emotional, functional, and physical. Each item is rated on a 5-point Likert scale ranging from 1 to 5, where 1 stands for “strongly agree”, 5 symbolizes “strongly disagree”, and 3 means “no opinion”. The global domain is presented separately, while a sum of the other scores and a mean score are calculated.

3 months after treatment(s), both LC and OPC/HPC patients were administered the University of Washington Quality of Life Questionnaire (UW-QOL), which consists of 12 single-question domains. Each domain has between 3 and 6 response options that are scaled evenly from 0 (worst) to 100 (best) according to the hierarchy of response. This questionnaire examines different domains such as appearance, activity, recreation, swallowing, chewing, speech, shoulder function, taste, salivation, mood and anxiety.

### Statistical analysis

Results are reported as value (%) and mean value ± standard deviation, as appropriate. Differences in the distribution of continuous variables between two independent groups were tested using the t-student test. Differences in the distribution between categorical variables were assessed through the chi square or fisher exact test, when appropriate. A two-sided *p* < 0.05 was considered significant. Statistical analysis was carried out using IBM SPSS Statistics software (version 28.0.1.0).

## Results

### Laryngeal cancer group (LC)

A total of 79 early-stage glottic cancers were included in the current study. Among them, 50.1% (40/79) underwent primary TOELS, while 49.9% (39/79) underwent primary TOLMS.

All patients alive included in the study completed the questionaries. The mean UW-QOL scores for specific domains reported by participants who completed the questionnaire were 1095 ± 246.9 for the exoscope group and 1170 ± 66.5 for the microscope group. No statistically significant difference was observed between the two groups (*p*-value = 0.37).

Post-operative VHI-10 scores between the two groups also did not show a statistically significant difference (*p*-value = 0.34).

The mean operative time was 69.5 ± 27.0 min in the exoscope group and 77.5 ± 35.7 min in the microscope group (*p*-value = 0.47). Hospitalization stay duration were similar between the two groups (*p*-value = 0.064).

In terms of margin status, superficial positive margins were found in 20.6% of patients in the TOELS group and 17.9% of patients in the TOLMS group. Meanwhile, deep positive margins were observed in 23.5% of patients in the TOELS group and 7.7% of patients in the TOLMS group. Both superficial and deep margins in the two groups showed comparable outcomes (*p*-value = 0.77 for the former and 0.059 for the latter). None of the patients underwent adjuvant RT. Data about the population is reported in Tables 1, 2.

**Table 1:** Demographic data regarding the laryngeal and oropharyngeal cancer groups of patients. Results are reported as value (%) and mean value ± standard deviation, as appropriate.

Laryngeal cancer group			*p*-value
	TOELS = 40 (50.1)	TOLMS = 39 (49.9)	
Age	71.7 ± 8.6	71.2 ± 12.3	0.73
Gender			
Male	31 (80.0%)	32 (80.0%)	0.95
Female	9 (20.0%)	7 (20.0%)	
Laryngoscore	4.9 ± 1.8	4.7 ± 1.8	0.69
pT classification			
pT1	38 (95.0%)	27 (69.2%)	0.025
pT2	2 (5.0%)	4 (10.2%)	
pT3	0 (0.0%)	5 (12.8%)	
Subsite			
Glottis	40 (100.0%)	31 (79.5%)	0.002
Supraglottis	0 (0%)	9 (20.5%)	
Oropharyngeal cancer group			
	TOELS = 16 (37.2)	(CT)RT = 27 (62.8)	
Age	71.5 ± 10.7	66.5 ± 10.9	0.18
Gender			
Male	14 (93.7%)	16 (62.9%)	0.031
Female	1 (6.3%)	11 (36.1%)	
P16 positivity	6 (37.5%)	21 (77.8%)	0.062
Stage p16+			
Stage I	5 (83.3%)	5 (23.8%)	0.99
Stage II	1 (16.7%)	16 (76.2%)	
Stage p16−			
Stage II	1 (14.3%)		0.99
Stage III	2 (28.6%)		
Stage IVA	3 (42.9%)	2 (40.0%)	
Stage IVB	1 (14.3%)	3 (60.0%)	

TOELS, transoral exoscopic laryngeal surgery; TOLMS, transoral laryngeal microsurgery; (CT)RT, (chemo)radiotherapy.

**Table 2:** Comparison of quality-of-life and surgical outcomes in laryngeal patients undergoing exoscope-assisted and microscopic-assisted surgical approaches. Hospitalization is analyzed for glottic patients only. Results are reported as value (%) and mean value ± standard deviation, as appropriate.

Variable	Treatment approach: number (%)		*p*-value
	TOELS = 40 (50.1)	TOLMS = 39 (49.9)	
UW-QOL	1095 ± 246.9	1170 ± 66.5	0.37
Preoperative VHI-10	1.7 ± 3.2	2.5 ± 3.2	0.041
Postoperative VHI-10	8.3 ± 10.2	10.5 ± 9.5	0.34
Delta VHI (post-pre)	7.0 ± 11.3	8.0 ± 10.4	0.61
Operative time (min)	69.5 ± 27.0	77.5 ± 35.7	0.47
Hospitalization (days)*	2.1 ± 0.4	2.5 ± 1.0	0.064
Superficial margin			
Positive	7 (20.6%)	7 (17.9%)	0.77
Negative	27 (79.1%)	32 (82.1%)	
Deep margin			
Positive	8 (23.5%)	3 (7.7%)	0.059
Negative	26 (76.5%)	36 (92.3%)	

(CT)RT, (chemo)radiotherapy; TOELS, transoral exoscopic laryngeal surgery; TOLMS, transoral laryngeal microsurgery; UW-QOL, University of Washington quality of life questionnaire; VHI-10, voice handicap index- 10.

### Oropharyngeal cancer group (OPC/HPC)

Included in the study were 43 cases of early-stage OPC/HPC. Among them, 37.2% (16/43) underwent a primary TOEOS, while 62.8% (27/43) received (CT)RT. All patients alive included in the study completed the questionaries. The mean UW-QOL scores for specific domains reported by participants who completed the questionnaire were 1078.1 ± 182.8 for the exoscope group and 1051.9 ± 148.2 for the (CT)RT group. No statistically significant difference was observed (*p*-value = 0.39). This is true also when considering global domains of the UW-QOL scores.

Post-treatment MDADI scores between the two groups did not show a statistically significant difference (*p*-value = 0.54). These results are summarized in Table 3.

**Table 3:** Comparison of quality-of-life outcomes in oropharyngeal patients undergoing exoscope-assisted surgery and (C)RT. Results are reported as value (%) and mean value ± standard deviation, as appropriate.

Variable	Treatment approach: number (%)		*p*-value
	TOEOS = 16 (37.2)	(CT)RT = 27 (62.8)	
UW-QOL (specific domains)	1078.1 ± 182.8	1051.9 ± 148.2	0.39
UW-QOL (global domains)	141.9 ± 70.6	138.8 ± 50.8	0.95
MDADI pre-treatment	91.3 ± 15.3	99.2 ± 3.7	0.028
MDADI post-treatment	79.7 ± 20.3	82.3 ± 7.6	0.54
Delta MDADI (pre-post)	11.6 ± 17.1	16.9 ± 7.8	0.067

(CT)RT, (chemo)radiotherapy; TOEOS, transoral exoscopic oropharyngeal surgery; UW-QOL, University of Washington quality of life questionnaire; MDADI, M.D. Anderson dysphagia inventory.

## Discussion

In recent decades, alterations in epidemiological patterns, the distinctive prognostic implications of HPV-associated cases, and advancements in treatment modalities have collectively contributed to a notable increase in the population of survivors of HNC (1). This article navigates through the multifaceted issues encountered by LC and OPC/HPC patients, emphasizing their profound impact on health related QOL. Beyond the conventional realms of pain and fatigue, HNC survivors grapple with intricate challenges such as oral dysfunction, voice impairment, and difficulties in speech and swallowing (28). Moreover, the long-term consequences of radiotherapy (RT), such as xerostomia, dysphagia, alterations in taste, and hypothyroidism, are well-documented in the literature (29). These challenges, often accompanied by social withdrawal and emotional distress, necessitate a comprehensive evaluation for informed treatment decisions (30). The importance of considering the QOL has recently gained prominence in the decision-making process for treating individuals with HNC (7, 31). Nevertheless, over the past few decades, the medical field has witnessed a surge in technological advancements aimed at mitigating the adverse effects of cancer treatments. One notable innovation is the VITOM 3D exoscope, which is gaining recognition as a promising option (32). Referring to a previous study we published, the article establishes that surgeries performed using VITOM 3D have demonstrated comparable outcomes to those reported in the existing literature (22, 33). However, before advocating the widespread adoption of a new technology that may improve certain aspects of treatment, it is crucial to ensure it does not cause harm.

The core inquiry of the study revolves around whether the VITOM 3D exoscope could indeed emerge as a valuable option for improving the QOL of LC and OPC/HPC patients when compared to well-established treatments.

### Laryngeal cancer group (LC)

TOLMS stands as a well-established surgical approach for T1-T2 LC (34). TOLMS is presented as a robust alternative to open-neck procedures and RT, offering compelling oncological results, if not superior, while reducing costs and achieving similar functional outcomes (35). We decided to exclusively incorporate surgically treated patients to facilitate the comparison of data concerning resection margins, operative time, and hospital stay, specific to TOLMS, recognized as the gold standard procedure for early to intermediate stage laryngeal malignancies. Additionally, given that within our institution, patients effected by early-stage laryngeal tumors and with a favorable predicted exposure are predominantly submitted to transoral surgery, we have opted to enhance the comparability between the two cohorts (TOELS and TOLMS) in terms of disease stage and exposure—the two pivotal factors profoundly influencing surgical outcomes. Consequently, we have chosen to juxtapose those treated with TOELS against their counterparts undergoing TOLMS. Such pertinent information would not have been ascertainable for patients subjected to radiation therapy. The surgical outcomes in the present study, including operating time, margin status, and hospital stay, closely align with those reported in the published literature (36). No statistically significant differences were observed between the group treated with a microscope and the group treated with an exoscope. In the laryngeal group, subjected to both TOLM and TOELS treatments, no postoperative complications were identified.

Moreover, the results from UW-QOL questionnaires failed to reveal any statistically significant differences, mirroring the lack of statistical distinction in pre-operative and post-operative VHI-10 scores. Notably, to the best of our knowledge, it's worth mentioning that there is a dearth of data on this specific aspect in the current literature.

These findings collectively suggest that both the TOLMS and TOELS represent valuable treatment options for laryngeal carcinoma, with comparable outcomes in terms of oncological efficacy, surgical proficiency, and QOL.

### Oropharyngeal cancer group (OPC/HPC)

Traditionally, OPC/HPC has been treated primarily with (CT)RT (37). However, over the past few decades, a less aggressive surgical alternative in the form of lateral oropharyngectomy has been gaining traction (38–40). However, direct comparisons of surgical and non-surgical approaches are limited in published literature, particularly with regard to functional outcomes (16).

Two case-control studies provide support for a swallowing-specific functional advantage associated with primary transoral robotic surgery (TORS) over primary chemoradiation in the treatment of OPC. In an unmatched case-control study included in this review, superior MDADI scores were reported among patients who underwent primary TORS compared to those treated with chemoradiation. The authors identified time-dependent, statistically significant differences in MDADI scores between the two treatment groups, favoring the primary surgical approach. Although no significant differences were observed in MDADI scores at the 3-month mark, patients treated with TORS exhibited significantly better scores at 6 and 12 months, suggesting improved long-term recovery following primary TORS compared to chemoradiation. These trends of enhanced swallowing-related QOL within the TORS group remained consistent when stratified by T-stage or oropharyngeal tumor subsite (16).

Our analysis aimed to discern whether there were differences in terms of QOL and dysphagia when assessed using MDADI. The results revealed no statistically significant differences between the two approaches. This suggests that both options hold value in the treatment of OPC/HPC. For these reasons, based on the findings in the literature and our own results, both the exoscope, robotic surgery, and (CT)RT demonstrate comparable scores. However, a more exhaustive exploration involves delving into the broader implications of incorporating emerging technologies into cancer treatment. Additional considerations need to be made by examining the costs, reproducibility, and learning curve associated with adopting such advanced tools. While our study provides valuable insights into the immediate outcomes, the long-term impact on QOL and potential complications arising from the use of the VITOM 3D exoscope warrant sustained investigation. Moreover, the cost-benefit analysis should not only consider the immediate clinical outcomes but also the economic feasibility and sustainability of incorporating advanced technologies into routine clinical practice.

Interestingly, despite the similar outcomes, a majority of patients tend to prefer surgery due to the perception of fewer “hidden costs” associated with this procedure. These “hidden costs” encompass intangible expenses such as travel time, travel distance, and missed work. Notably, our findings indicated that these intangible expenses were higher among patients who underwent radiation therapy compared to those who underwent transoral endoscopic resections (41).

The current study has several limitations that need to be acknowledged. Firstly, like every study investigating quality of life through a questionnaire, the results are subject to the intrinsic limitations of the exploring tool. A notable limitation of the MDADI questionnaire, encountered during its administration, is its emphasis on a few surgical-objective functional outcomes. Many questionnaire items primarily address the psychological and emotional aspects of the patient, leading to an alteration of the overall questionnaire results due to the inherent structure of the questionnaire itself.

Additionally, factors such as the surgeon's expertise with the new exoscope technology, potential biases associated with the retrospective nature of the study, subjectivity in responses to the questionnaires, the relatively small number of enrolled patients, and the nature of the questionnaires themselves—more inclined to assess psychological and emotional aspects than specific functional outcomes resulting from treatments—should be considered.

To gain a more comprehensive understanding of these parameters, further prospective comparative studies are warranted.

In the domain of Head and Neck surgery, the application of 3D exoscopes presents potential advantages. Assessments of QOL, voice quality, and dysphagia, demonstrate comparable outcomes between traditional treatment modalities and transoral interventions employing the 3D exoscope. Parameters such as operative time, duration of hospitalization and margin status demonstrate overlapping characteristics between the cohort treated with the exoscope and those treated with the microscope in the LC group. Before conducting a survival analysis involving a new technique or therapy, it is important to verify that such a new approach is both effective and safe for patients. For this reason, the purpose of our study is not to perform a comparative survival analysis between two treatment modalities. Instead, we aimed to evaluate the quality of life of patients to understand whether the use of the new exoscope impacts the quality of life compared to gold standard treatments (radiochemotherapy for oropharyngeal tumors and TOLM for laryngeal tumors). Additionally, we sought to verify that this approach is technically effective by comparing outcomes such as margin status, hospitalization, and duration of the procedure in the operating room compared to a treatment considered gold standard (TOLM for laryngeal tumors). Certainly, conducting a survival analysis among different treatment methods is an interesting topic to explore, but it requires adequate follow-up (generally a median of at least 2 years). However, before doing so, it is important to verify that a treatment method is safe, effective, and does not impact the quality of life of patients. In light of the current study, we are collecting data for a subsequent study regarding survival outcomes of patients with LC treated with TOLMS vs. TOELS and with (CT)RT vs. TOELS.

Nevertheless, it is worth of mention that the present study was conducted at a singular center. Further investigation, encompassing larger patient cohorts and involving multiple centers, is imperative to validate these findings and assess the generalizability of the results.

## Data Availability

The raw data supporting the conclusions of this article will be made available by the authors, without undue reservation.
